# Correction: One pot green preparation of *Seabuckthorn* silver nanoparticles (SBT@AgNPs) featuring high stability and longevity, antibacterial, antioxidant potential: a nano disinfectant future perspective

**DOI:** 10.1039/d0ra90123g

**Published:** 2020-11-26

**Authors:** Thiyagarajan Kalaiyarasan, Vijay K. Bharti, O. P. Chaurasia

**Affiliations:** Defence Institute of High Altitude Research (DIHAR), DRDO Leh-Ladakh-194101 J&K India vijaykbharti@rediffmail.com +91-172-2638500 +91-172-2638900

## Abstract

Correction for ‘One pot green preparation of *Seabuckthorn* silver nanoparticles (SBT@AgNPs) featuring high stability and longevity, antibacterial, antioxidant potential: a nano disinfectant future perspective’ by Thiyagarajan Kalaiyarasan *et al.*, *RSC Adv.*, 2017, **7**, 51130–51141, DOI: 10.1039/c7ra10262c.

The authors regret that an incorrect versions of Fig. 1(c), 3, 7 and 9(b) were included in the original article. The correct versions of these figures are presented below.

**Fig. 1:**
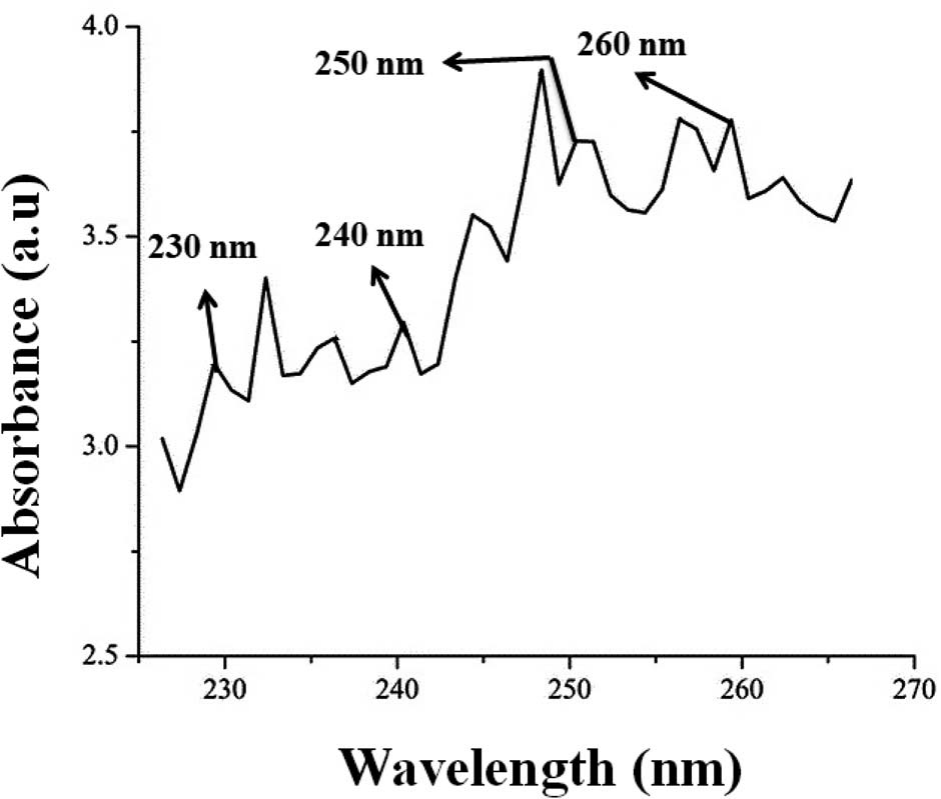
(c) UV spectra for leaf extracts speak shown at 230 nm, 240 nm, 250 nm and 260 nm wavelengths indicates the presence of phenol compounds.

**Fig. 3:**
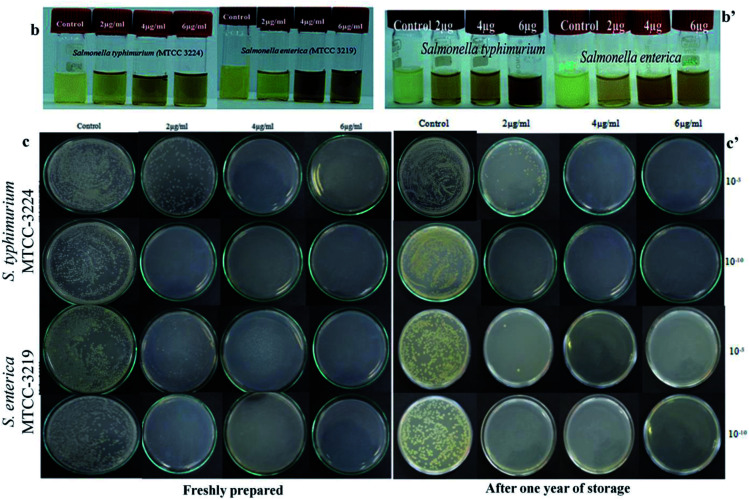
(b and b′) Vial image showing turbidity assay towards *salmonella* Sps and Petri plate image showing colonies of *salmonella* Sps incubated on BHI agar obtained from cultivated suspension with SBT@AgNPs. (c and c′) Quantitative evaluation of the antimicrobial ability of freshly prepared and after one year stored SBT@AgNPs by counting the colonies (CFU) grown on BHI agar plates. The original concentration of cells are about ∼105 CFU mL^−1^. Antibacterial efficiency was calculated following equation: antibacterial rate (%) = (*N*_control_ − *N*_sample_)/*N*_control_ × 100 mean values and standard deviation are calculated from four independent experiments.

**Fig. 7:**
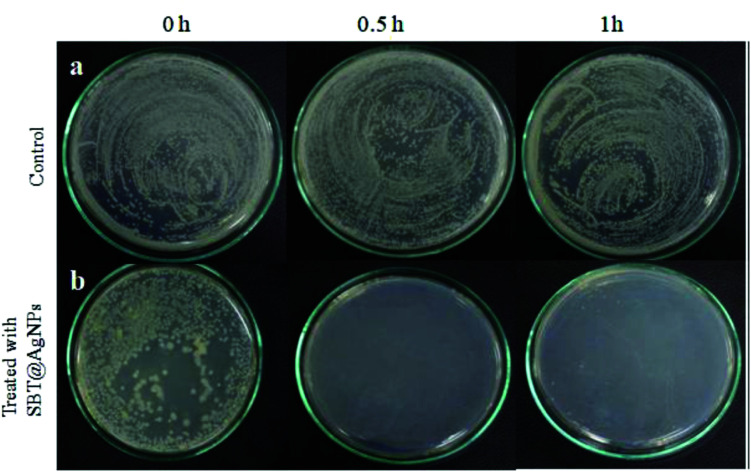
Investigation of antibacterial activity against bacterial cocktail on routine infection-control measures and environmental decontamination using SBT@AgNPs in the day to day life. (a) Untreated (control), (b) treated with SBT@AgNPs (6 μg mL^−1^).

**Fig. 9:**
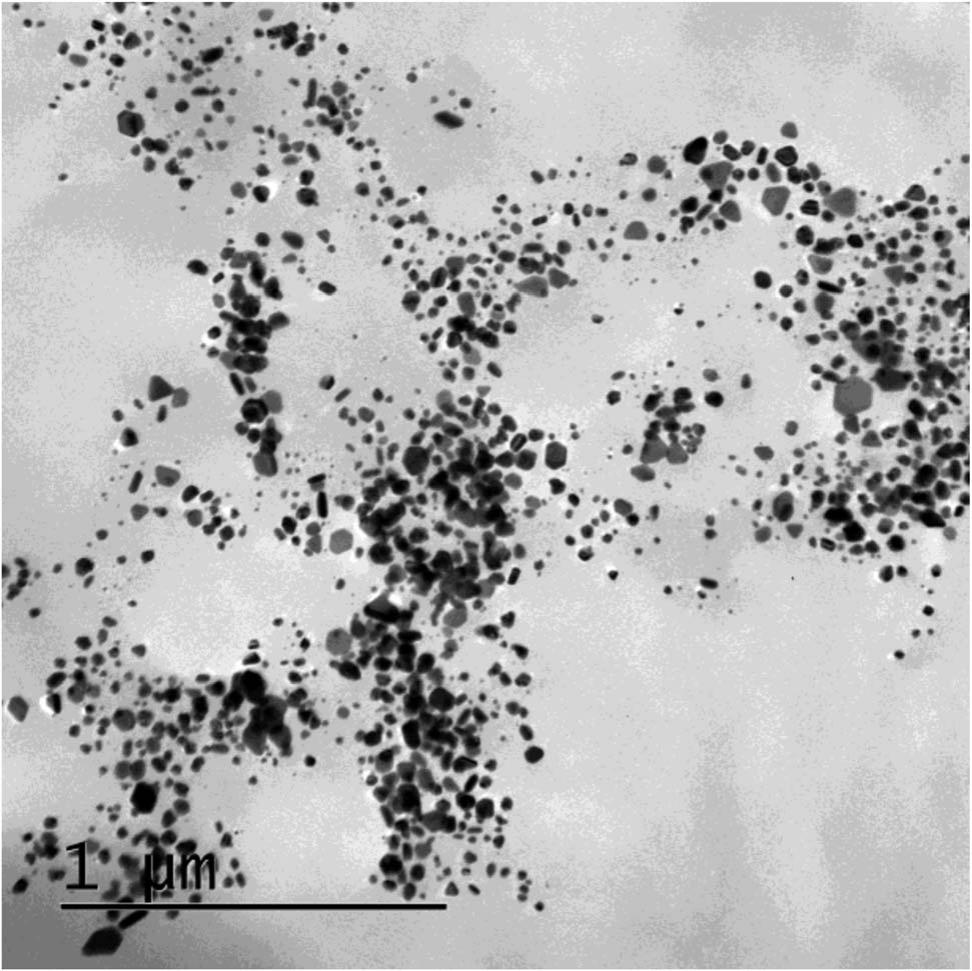
(b) TEM image of SBT@AgNPs after one year of synthesis.

The Royal Society of Chemistry apologises for these errors and any consequent inconvenience to authors and readers.

## Supplementary Material

